# Somatic gene mutations in the motor cortex of patients with sporadic amyotrophic lateral sclerosis

**DOI:** 10.1093/brain/awaf460

**Published:** 2025-12-11

**Authors:** Óscar González-Velasco, Rosanna Parlato, Rüstem Yilmaz, Lorena Decker, Sonja Menge, Axel Freischmidt, Xiaoxu Yang, Nikshitha Tulasi, David Brenner, Peter M Andersen, Karin M E Forsberg, Johannes C M Schlachetzki, Benedikt Brors, Lena Voith von Voithenberg, Jochen H Weishaupt

**Affiliations:** Department of Applied Bioinformatics, German Cancer Research Center (DKFZ), Heidelberg 69120, Germany; Division of Neurodegeneration, Department of Neurology, Mannheim Center for Translational Neurosciences, Medical Faculty Mannheim, Heidelberg University, Mannheim 68167, Germany; Division of Neurodegeneration, Department of Neurology, Mannheim Center for Translational Neurosciences, Medical Faculty Mannheim, Heidelberg University, Mannheim 68167, Germany; Division of Neurodegeneration, Department of Neurology, Mannheim Center for Translational Neurosciences, Medical Faculty Mannheim, Heidelberg University, Mannheim 68167, Germany; Department of Neurology, Ulm University, Ulm 89081, Germany; Department of Neurology, Ulm University, Ulm 89081, Germany; Department of Neurology, Ulm University, Ulm 89081, Germany; Department of Human Genetics, University of Utah, Salt Lake City, UT 84111, USA; Division of Neurodegeneration, Department of Neurology, Mannheim Center for Translational Neurosciences, Medical Faculty Mannheim, Heidelberg University, Mannheim 68167, Germany; Department of Neurology, Ulm University, Ulm 89081, Germany; Department of Clinical Sciences, Neurosciences, Umeå University, Umeå 90187, Sweden; Department of Clinical Sciences, Neurosciences, Umeå University, Umeå 90187, Sweden; Department of Neurosciences, University of California, San Diego, La Jolla, CA 92093, USA; Department of Applied Bioinformatics, German Cancer Research Center (DKFZ), Heidelberg 69120, Germany; Department of Applied Bioinformatics, German Cancer Research Center (DKFZ), Heidelberg 69120, Germany; Division of Neurodegeneration, Department of Neurology, Mannheim Center for Translational Neurosciences, Medical Faculty Mannheim, Heidelberg University, Mannheim 68167, Germany; Department of Neurology, Ulm University, Ulm 89081, Germany

**Keywords:** amyotrophic lateral sclerosis, neuronal somatic mosaicism, somatic variants, excitatory neurons

## Abstract

Amyotrophic lateral sclerosis (ALS) is characterized by the progressive degeneration of cortical and spinal motor neurons. Mendelian germline mutations often cause familial ALS (fALS) but only approximately 10% of sporadic ALS cases (sALS).

We leveraged DNA and single-cell RNA sequencing data from autopsy tissue to explore the presence of somatic mosaic variants in sALS cases.

Deep targeted panel sequencing of known ALS disease genes in motor cortex tissue revealed an enrichment of low allele frequency variants in sALS, but not in fALS with an identified monogenic cause. *In silico* analysis predicted increased pathogenicity of mosaic mutations in various known ALS mutational hot-spots. In particular, we identified the somatic FUS variant p.E516X, located in an established hotspot for germline ALS mutations, which leads to nucleo-cytoplasmic mislocalization and aggregation typical for ALS FUS pathology. Additionally, we performed somatic variant calling on single-cell RNA-sequencing data from sALS tissue and revealed a specific accumulation of somatic variants in excitatory neurons, reinforcing a neuron-autonomous disease initiation.

Collectively, this study indicates that somatic mutations within the motor cortex, especially in excitatory neurons, may contribute to sALS development.

## Teaser

González-Velasco *et al.* show that somatic mutations in known ALS genes are enriched in the motor cortex of patients with sporadic ALS, particularly in excitatory neurons. These variants may contribute to disease initiation, providing insight into pathogenic mechanisms beyond inherited germline mutations.

## Introduction

Amyotrophic lateral sclerosis (ALS) is an adult-onset neurodegenerative disease characterized clinically by degeneration primarily of motor neurons eventually leading to respiratory failure and death.^1,2^ Only about 5%–10% of European ALS patients report a positive family history for the disease (fALS),^3^ usually with an autosomal dominant mode of inheritance, whereas most ALS patients report a family history that is unremarkable for ALS (sALS). Rare cases of germline *de novo* mutations, in particular in *FUS* and *SOD1* as a cause of sALS, have been reported.^4,5^ However, whereas even in a considerable proportion (∼50%) of fALS cases screening for a germline mutation in blood DNA remains inconclusive, conventional genetic testing turns out negative in approximately 90% of the people with sALS.^6^ On the other hand, twin studies point to a considerably higher contribution of genetic factors to ALS pathogenesis than explained by the frequency of Mendelian mutations.^7^

Insights from neuropathology, but also progression of clinical symptoms, suggest that ALS pathology starts focally as a proteinopathy^8,9^ and then spreads contiguously within the central nervous system over time.^10^ Biological and clinical data support the notion that a prion-like mechanism with focal initiation may be involved in spreading toxic RNA and protein species including misfolded SOD1, TDP43 and FUS.^11,12^ Such a hypothesis entails that even a small number of pathologically altered cells may be sufficient to initiate the focal development of ALS pathology, with expanding motoneuronal demise, and eventually leading to clinically progressive manifest muscle weakness.^13^ Studies in chimeric mice transgenic for fALS *SOD1* mutations suggest a neuron-autonomous initiation of ALS.^14^ Furthermore, a combination of multiple genomic alterations may increase the risk of ALS development.^15^ Considering these prior data, here we tested the hypothesis that somatic mosaic mutations could account for a proportion of sALS of unknown monogenic origin, and aimed at the identification of cellular populations affected by these variants. To this end, we combined deep targeted sequencing of ALS-related genes, targeted amplicon sequencing and single-cell RNA-sequencing of post-mortem motor cortex tissue followed by a proof-of-concept pathogenic validation of selected mosaic mutations in the ALS gene *FUS*. This study shows that somatic mutations in ALS-related genes may play a role in sALS pathogenesis, and that within the motor cortex excitatory neurons are more prone to accumulate somatic mutations.

## Materials and methods

### Patient cohort

Fresh frozen autoptic human precentral gyrus and spinal cord tissues of donors with ALS and control donors were provided by the ALS Brain Bank at Umeå University in Sweden and the Netherlands Brain Bank (Supplementary Table 1 and Supplementary material, 'Materials and methods' section). Patients, who donated tissue to the ALS Brain Bank at Umeå University and the Netherlands Brain Bank, provided written informed consent for the molecular genetic research reported in this study. The control cases were characterized by both clinical and pathological criteria, according to the latest international diagnostics criteria as specified in https://www.brainbank.nl/brain-tissue/diagnostics/. Neuropathological examination did not change the clinical diagnosis in any of the Netherlands Brain Bank or Swedish cases.

### Targeted deep sequencing

A 50 ng sample of genomic DNA was used for library preparation and target enrichment (Supplementary material, 'Materials and methods' section). The list of targeted ALS genes is provided in Supplementary Table 2. Next-generation sequencing was performed at the Sequencing Core Facility of the German Cancer Research Center (Supplementary material, 'Materials and methods' section). The samples were sequenced on a HiSeq 4000 instrument (Illumina) by paired-end sequencing of 100 base pairs targeting a coverage of ≥2000-fold. Per sample ∼10–20 million reads were obtained. A schematic overview of the analysis workflow is provided in Supplementary Fig. 1.

### Functional validation of variants

The coding sequence of human wild-type *FUS* (NM_004960.4) was cloned into BglII and KpnI sites of pCMV-Myc-*N* (Clontech Laboratories). Whereas FUS p.R495X was already available, other variants were introduced by site-directed mutagenesis as described in the manual of the QuikChange II Site-Directed Mutagenesis Kit (Agilent Technologies). Respective oligonucleotides for *FUS* mutagenesis are listed in Supplementary Table 3. All sequences were verified by Sanger sequencing.

HEK293 cells were transfected with plasmid DNA using calcium phosphate precipitation with minor modifications. At 24 h post-transfection, immunocytochemistry was performed as recently described^16^ using primary mouse anti-myc (1:500, Cell Signaling Technology, 2276) and secondary donkey anti-mouse-647 antibodies (1:500, Thermo Fisher Scientific, A-31571). Images were acquired on a Zeiss LSM 980 confocal microscope and analysed using ImageJ.

### Single nucleotide variant calling from single-cell RNA sequencing data

Fastq files from a public single-cell RNA sequencing dataset^17^ were processed using cellranger version 8.0.1, and human genome reference version GRCh38. We then used SComatic for somatic variant calling (Supplementary material, 'Materials and methods' section), annotations of groups of cells were defined using the data’s original cell types. In total, after pre-processing we analysed around 720 000 cells [number of samples: fALS = 5, familial frontotemporal lobar degeneration (fFTLD) = 5, control = 15, sALS = 13, sporadic FTLD (sFTLD) = 12].

### Statistical analysis

Statistical analyses of variant occurrence between disease categories were performed using negative binomial generalized linear models (GLM) including disease category (sALS, fALS, control) as the main predictor and sex, age, origin of patient and total coverage as covariates to control for potential confounding effects (Supplementary material, 'Materials and methods' section).

## Results

### Higher somatic variant burden in ALS-related genes in sALS patients identified by targeted deep sequencing

**Figure 1:**
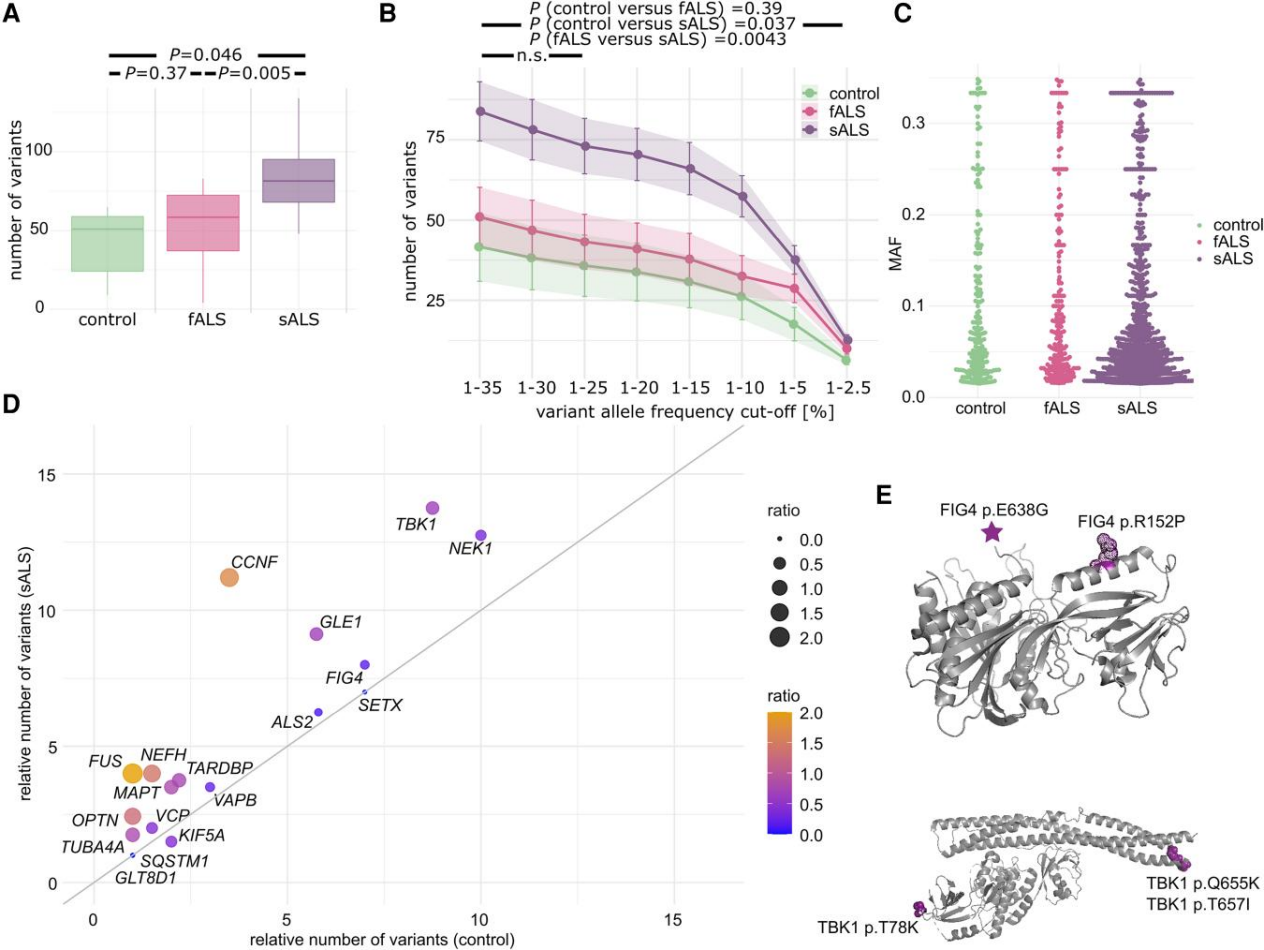
**Somatic variant identification by targeted deep sequencing.** (**A**) Number of variants with an allele frequency (AF) range of 35% > AF > 1.5% detected per sample in all genes of the targeted panel. (**B**) Number of variants detected per variant AF for the different groups of samples. Enrichment test by using a generalized linear model with covariates (total coverage, age, sex, origin) and adjusted for multiple testing by FDR approximation. *P*-values are provided for testing an AF range of 0.35 < AF < 0.015. *P*-values were non-significant (n.s.) for the AF range 0.35 < AF < 0.25 for any of the conditions compared. (**C**) AF distribution of variants by group of samples. (**D**) Frequency of number of variants per sample in the indicated genes of the targeted panel in the control group versus the sALS group coloured by the ratio of the number of variants between sALS and control samples. (**E**) Exemplary protein positions of SNVs detected in ALS samples displayed on the protein structures of FIG4 (pdb: 7K1W) and TBK1(pdb: 6NT9). fALS = familial amyotrophic lateral sclerosis; FDR = false discovery rate; sALS = sporadic amyotrophic lateral sclerosis; SNV = single nucleotide variant.

To detect germline and somatic mutations in sALS brains by deep sequencing, we analysed motor cortex DNA of nine individuals diagnosed as sALS patients, four familial ALS (fALS) cases, which carried germline mutations in fALS genes (*SOD1*, *TBK1*, *NEK1* and *C9ORF72*), and six control cases (Supplementary Fig. 1 and Supplementary Table 1). Single nucleotide variants (SNVs) were detected by combining error-correction based on unique molecular identifiers and calling and integration from multiple variant callers (Supplementary Fig. 2). To identify somatic mosaic mutations by targeted deep sequencing, we specifically selected variants with low variant allele frequency (VAF). The overall relative distribution of SNVs and small insertions and deletions in the different genomic regions, e.g. exonic, 3’UTR, 5’UTR, intergenic, was similar between controls, fALS and sALS patients (Supplementary Figs 3–5). Interestingly, we observed an increase in the total number of somatic variants in sALS patients (μ = 83.3, σ = 25.7) compared to controls (μ = 41.6, σ = 24.03, *t*-test *P*-value = 0.046) (Fig. 1A and Supplementary Fig. 3D). No significant difference was noted between fALS and controls (*P*-value = 0.37). An analysis of the number of SNVs by allele frequency (AF) showed an overall increase in the number of SNVs in the genes of interest for AF between 1.5% and 35% in sALS cases, which was driven by somatic variants (Fig. 1B and C). Whereas most of the genes known to play a role in fALS showed an increase in the number of variants in sALS patients versus controls, we observed a specifically strong enrichment in the number of SNVs in *VAPB*, *MAPT*, *FUS*, *NEFH*, *CCNF*, *NEK1* and *TBK1*, for some of which the number of somatic SNVs almost doubled (Fig. 1D, Supplementary Table 4 and Supplementary material). SNVs detected in these genes are positioned in different protein regions, e.g. α-helical chains of the proteins, which may impact protein function (Fig. 1E). In summary, we identified an overall increased somatic variant burden and several pathogenic somatic mutations in ALS-associated genes in sALS patients, indicating somatic mosaic variants as potential contributors to sALS development.

### Functional validation of somatic mosaic variants in FUS detected in sALS patients

**Figure 2:**
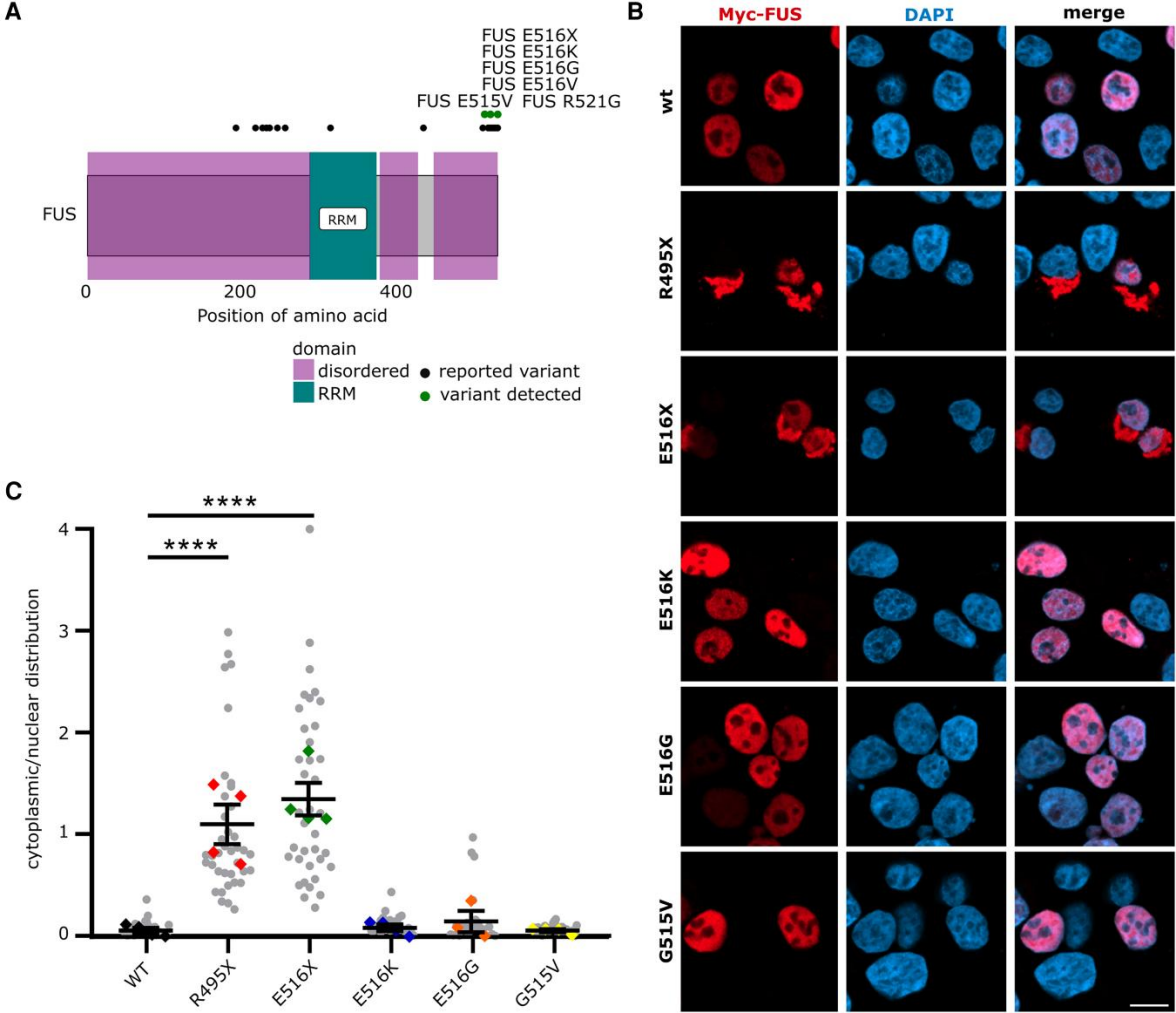
**Functional validation of variants in FUS observed as somatic mosaic variants in sALS patients.** (**A**) FUS protein structure with functional domains and known SNVs and SNVs detected in this study. (**B**) Cellular localization of wild-type and mutant FUS in cultured HEK293 cells. Scale bar = 10 μm. (**C**) Ratio of cytoplasmic to nuclear localization of FUS protein for the different FUS variants. sALS = sporadic amyotrophic lateral sclerosis; SNV = single nucleotide variant.

To better understand the potential functional relevance of somatic mutations in ALS genes in the motor cortex, as a proof-of-principle, we focused on *FUS* variants detected here in a mosaic pattern. *FUS* represents an exemplary ALS gene with well-defined neuropathology in post-mortem tissue and in *in vitro* models.^18,19^ Hence, we reasoned to functionally validate the variants predicted to occur in a somatic mosaic pattern in FUS (Fig. 2A), and we analysed the cellular localization of the protein in cultured cells (Fig. 2B). Mutant FUS proteins carrying the four variants were cloned and transiently overexpressed in HEK293 cells in comparison to wild-type controls and a known pathogenic fALS variant FUS p.R495X. Whereas wild-type FUS was localized in the nuclei in a well-defined pattern, the FUS p.R495X variant showed cytoplasmic aggregation (Fig. 2B and C). The FUS p.E516X variant, which we detected in ALS patients in a somatic mosaic pattern, was also localized in aggregated foci in the cytoplasm, losing its nuclear localization. The cellular localization of FUS p.E516K, FUS p.E516E and FUS p.G515V was only minimally affected under these experimental conditions, which is in line with pathogenic effects of FUS variants even in the absence of cytoplasmic aggregation.^20^

### Increased somatic variant burden in excitatory neurons identified by variant calling from single-cell sequencing data

**Figure 3:**
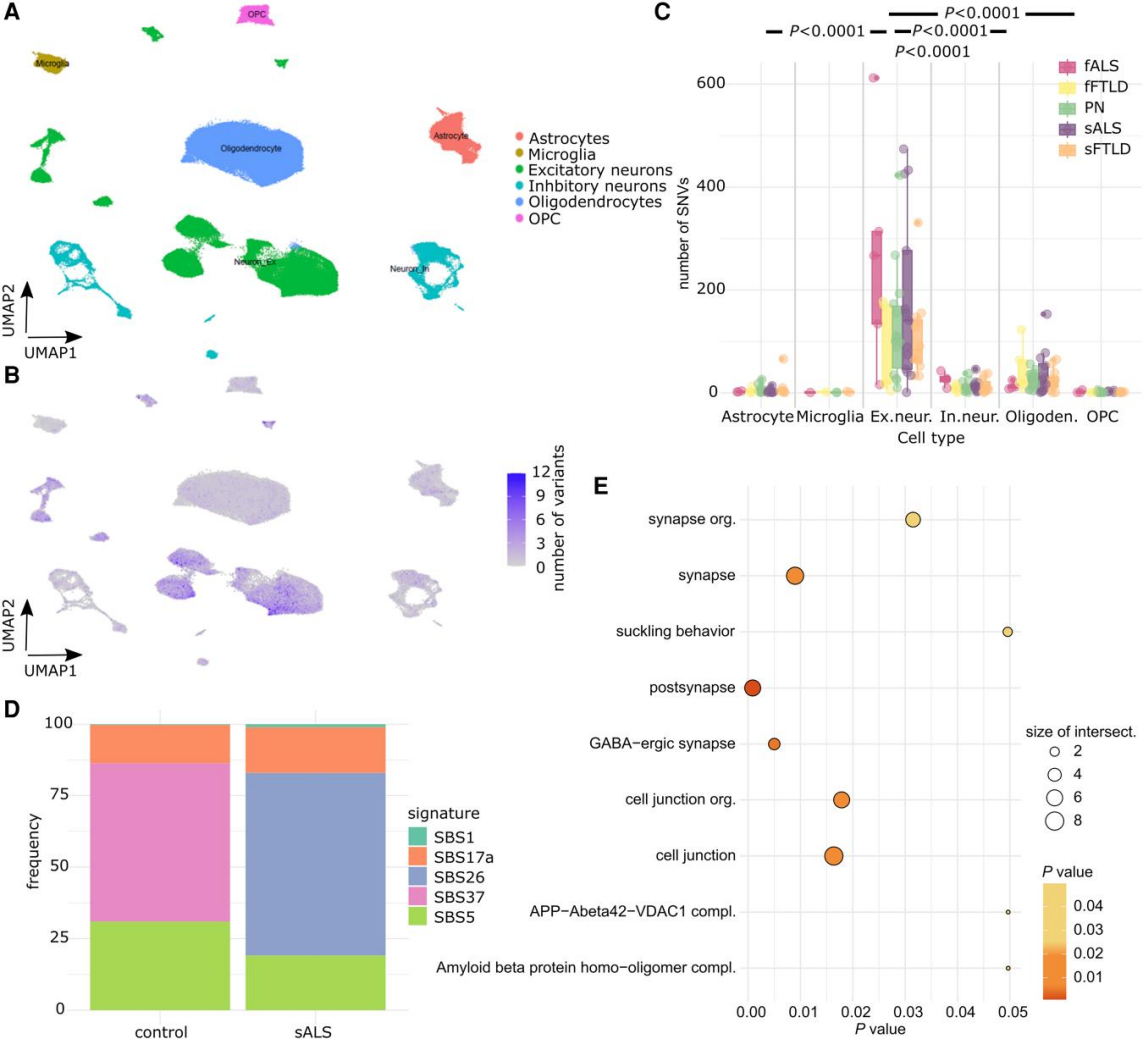
**Somatic variant calling from single-cell RNA sequencing data.** (**A**) Dimension reduction and visualization of single-cell RNA expression levels of clusters of cells by UMAP of a dataset with sALS, sFTLD and annotation of cell types. (**B**) UMAP of cell populations with the colour indicating the number of all (coding and non-coding) somatic variants detected by SComatic per cell. (**C**) Distribution of number of variants per cell type and disease status. Pairwise statistical analysis was performed by using a generalized linear model corrected for multiple testing and number of cells and shown exemplarily for sALS samples. (**D**) Relative fraction of DNA damage-related single base substitution signatures in controls in comparison to sALS samples. (**E**) Gene sets enriched for SNVs in excitatory neurons. fALS = familial amyotropic lateral sclerosis; OPC = oligodendrocyte precursor cells; sALS = sporadic amyotrophic lateral sclerosis; sFTLD = sporadic frontotemporal lobar degeneration; SNV = single nucleotide variant; UMAP = Uniform Manifold Approximation and Projection.

Considering somatic mutations a causative factor for ALS, the respective cells harbouring pathogenic somatic mutations would represent the starting point of the disease. Therefore, the preferentially affected cell type is of interest. To further understand the cell type-specific distribution of somatic mosaic variants in the motor cortex, we performed somatic variant calling in single-cell RNA-sequencing data from publicly available cohorts of sALS and fALS, sFTLD and fFTLD and control samples using SComatic (Fig. 3 and Supplementary Table 5).^17^ In total, we processed around 720 000 annotated cells.

Our analysis revealed that excitatory neurons were the cell type with the highest mutational burden for diverse ALS diagnoses (Fig. 3A–C). Two clusters of subpopulations of excitatory neurons were identified, in which the numbers of SNVs were especially high (clusters 5 and 9; Supplementary Fig. 6). These clusters were mainly composed of sALS cells, revealing a selective high vulnerability of these cells to accumulate SNVs.

By using all somatic variants detected in the excitatory neurons, we computed mutational signature profiles per condition. We observed a trend towards an enrichment of DNA damage signatures, e.g. SBS26 resulting from defective DNA mismatch repair, in sALS patients compared with controls (Fig. 3D). Gene set enrichment analysis showed an increase in somatic SNV burden in genes related to synapse and cell junction organization in sALS patients (Fig. 3E).

In summary, we identified an enrichment of somatic mutations predominantly in excitatory neurons, with an association to DNA damage repair signatures and cell junction and synapse organization, suggesting that altered DNA repair mechanisms may contribute to sALS pathogenesis.

## Discussion

Here, we show that sporadic, but not monogenic ALS caused by germline mutations, is linked to somatic mutations in the motor cortex. We also demonstrate ALS-typical pathology of FUS protein harbouring a specific mosaic mutation in sALS patients. Respective mutations usually disturb the nuclear localization sequence of the protein, leading to nucleocytoplasmic redistribution and cytoplasmic aggregation,^21^ accompanied by nuclear loss-of-function effects.^22^ We observed a robust cytoplasmic mislocalization and deposition of the FUS p.E516X mutant protein found in the mosaic state (7% allele frequency in an sALS patient) in cultured cells. Notably, if found as a germline mutation, the p.E516X would have been classified as ALS-causative. Moreover, analysis of single-cell transcriptomic data revealed an increased burden of somatic mutations in human motor cortex neurons, supporting a neuronal origin for sALS and showing that neurons are more prone to accumulate somatic mutations than other brain cell types.^23^ Excitatory neurons show an even higher enrichment of mosaic mutations than inhibitory neurons, in accordance with the view that sALS starts in excitatory neurons.^24^

The data support a pathogenic role of low-frequency, somatic mutations in sALS patients. In line with the hypothesis of multiple factors accounting for sALS onset and progression by the dysregulation of converging and common pathways,^25^ a population-based modelling study provided evidence that the development of ALS is a multistage process, involving six steps in the case of sporadic patients.^26^ The occurrence of multiple somatic mutations in the same and different ALS genes may represent one of these steps. Notably, in several sALS motor cortices, we detected somatic variants in more than one ALS gene, suggesting a poly- or oligogenic mosaic origin. It remains to be shown whether the different variants detected in an individual arose from the same cell or different cells in a ‘colony’ or even in cell types that could communicate to instigate pathology. The age-related accumulation of variants in neurons and genomic instability as an effect of defective DNA damage response should be considered as an sALS pathomechanism.^27,28^ Many of the known ALS-related genes impair DNA damage repair directly or indirectly.^29^ Thus, an accumulation of variants in DDR-related single-base substitution signatures as observed for excitatory neurons might contribute to the development of sALS.

The phenotype of ALS differs considerably between individuals with regard to site and age of onset and speed of progression. Somatic mutations, besides potentially playing a role in disease initiation, could also act as disease modifiers and partially account for phenotypic diversity. In particular, the site of onset might be partially determined by stochastically occurring variants that are restricted to specific regions of the CNS or even single cells. This would be compatible with the hypothesis that a small number of affected cells may be sufficient to instigate focal pathology, followed by contiguous systemic spreading of the disease over time.^10-13^

In summary, this study sheds new light on the origin of sALS. In perspective, these findings may have important implications for therapeutic design, because the identification of mosaic mutations could render respective patients suitable for gene-specific interventions, based on already successfully adopted antisense-oligonucleotides and small-interfering RNAs.^30^ A prerequisite will be the development of sensitive procedures for the diagnosis of ALS and for the reliable identification of mosaic mutations in biofluids from ALS patients.

## Supplementary Material

awaf460_Supplementary_Data

## Data Availability

The raw sequencing data will be made available at the European Genome-Phenome Archive (EGAS00001008104).
